# Molecular Evidence of Transmission of Influenza A/H1N1 2009 on a University Campus

**DOI:** 10.1371/journal.pone.0168596

**Published:** 2017-01-06

**Authors:** Ramandeep Kaur Virk, Vithiagaran Gunalan, Hong Kai Lee, Masafumi Inoue, Catherine Chua, Boon-Huan Tan, Paul Anantharajah Tambyah

**Affiliations:** 1 Department of Medicine, National University of Singapore, Singapore; 2 Bioinformatics Institute, Agency for Science, Technology and Research, Singapore; 3 Department of Laboratory Medicine, National University Hospital, Singapore; 4 Experimental Therapeutics Unit, Agency for Science, Technology and Research, Singapore; 5 University Health Centre, National University of Singapore, Singapore; 6 Detection & Diagnostics Laboratory, DSO National Laboratories, Singapore; Sidra Medical and Research Center, QATAR

## Abstract

**Background:**

In the recent years, the data on the molecular epidemiology of influenza viruses have expanded enormously because of the availability of cutting-edge sequencing technologies. However, much of the information is from the temperate regions with few studies from tropical regions such as South-east Asia. Despite the fact that influenza has been known to transmit rapidly within semi-closed communities, such as military camps and educational institutions, data are limited from these communities.

**Objectives:**

To determine the phylogeography of influenza viruses on a university campus, we examined the spatial distribution of influenza virus on the National University of Singapore (NUS) campus.

**Methods:**

Consenting students from the NUS who sought medical attention at the UHC provided two nasopharyngeal swabs and demographic data. PCR was used for detection of influenza viruses. 34 full-genomes of pH1N1/09 viruses were successfully sequenced by Sanger method and concatenated using Geneious R7. Phylogenetic analysis was conducted using these 34 sequences and 1518 global sequences. Phylogeographic analysis was done using BaTS software and Association index and Fitch parsimony scores were determined.

**Results:**

Integrating whole genome sequencing data with epidemiological data, we found strong evidence of influenza transmission on campus as isolates from students residing on-campus were highly similar to each other (AI, P value = 0.009; PS, P value = 0.04). There was also evidence of multiple introductions from the community.

**Conclusions:**

Such data are useful in formulating pandemic preparedness plans which can use these communities as sentinel sites for detection and monitoring of emerging respiratory viral infections.

## Introduction

Molecular data have been instrumental in studying the origin as well as persistence and migration of influenza viruses [1,2]. In addition, molecular studies provide detailed virus characteristics, such as type, subtype, lineage, virulence and drug resistance markers. They also provide insights into the pathways of viral transmission and help in understanding the phylogeography of influenza viruses when integrated with epidemiological data [3]. A better integration of molecular and epidemiological data may (i) help unfold the dynamics of spatial transmission, (ii) provide insights into the global transmission patterns of influenza viruses and the existence of source populations, and (iii) help public health authorities to plan more targeted intervention strategies. On a global level, in an event of impending pandemic, a better understanding of transmission patterns of novel or emerging viruses may help public health authorities to develop effective pandemic preparedness plans.

In recent years, molecular epidemiology has been applied to emerging influenza viruses, including pandemic H1N1 2009 (pH1N1/09) and H7N9 [4]. Although there are available data on the spatial and temporal transmission dynamics of influenza at global and national levels [5–10], transmission dynamics have not been thoroughly explored at the level of individual communities, especially in semi-closed settings such as universities or military camps. A better understanding of transmission dynamics in localized communities helps to identify foci of influenza transmission for more targeted interventions, such as school closure or household prophylaxis [11]. An examination of the spatial diffusion of pH1N1/09 virus in a localized community of the University of California found that although there was intra-campus transmission, there was no significant association of on-campus residence with clustering of similar viral strains [12].

We undertook this study to identify distinctive epidemiological factors associated with transmission of influenza on a university campus and to explore the molecular epidemiology of influenza in a university setting. We found that 79% (11/14) of the strains that formed clusters on phylogenetic analysis were from students staying on-campus (p <0.05), which suggests that a significantly higher proportion of the influenza was transmitted within campus than was introduced from sources outside of the university. This has tremendous implications on response to future pandemics. For instance, if the majority of influenza is found to be transmitted on campus, i.e. there is substantial virus clustering on campus and the influenza strains isolated are proven by molecular epidemiology to be closely linked, then, possible closure of large institutions *or* on-campus intervention methods to halt influenza transmission, such as, quarantine of hostels, cancellation of lectures, shut-down of canteen areas, closure of facility areas or ring prophylaxis may be important and useful strategies. If on the other hand, the majority of influenza strains are distinct and there is no evidence of clustering, suggesting importation from contacts off campus, then the major intervention strategies would be very different.

## Materials and Methods

### Samples

The study was approved by National University of Singapore (NUS) Institutional Review Board (reference number, 06–156; approval number, NUS-282) and written informed consent was obtained from the participants prior to sample and data collection. Two nasopharyngeal swabs and demographic data (age, gender, citizenship status, smoking status, residence address, and contact history) were collected from students and staff presenting with influenza-like illness (ILI) to the university health centre at the NUS [13]. Influenza infection was confirmed by PCR. Details of this cohort have been described previously [14].

### Virus sequencing

RNA was extracted using QIAamp Viral RNA mini kit (Qiagen, Inc., Valencia, CA, USA) according to manufacturer’s instruction. Reverse-transcription of vRNA was carried out using SuperScript First-Strand Synthesis System for RT-PCR (Invitrogen Corporation, CA, USA) and Uni 12 primer (5’AGCRAAAGCAGG3’) [15]. Full-length genes were obtained by performing PCR using primers described previously [15–17], and with primers obtained from the WHO Collaborating Centre for Reference and Research on Influenza in Melbourne (Dr. TBoon-Huan Tan, personal communication). Sanger sequencing was performed with ABI Prism Big DyeTerminator (ThermoFisher Scientific Inc.). The assembly and editing of raw sequence data were done using SeqMan (DNASTAR, Lasergene Version 7, Madison, USA). The sequences were deposited in GenBank and can be retrieved from the National Center for Biotechnology Information’s (NCBI) Influenza Virus Resource (http://www.ncbi.nlm.nih.gov/genomes/FLU/FLU.html).

### Preliminary analysis for related transmissions based on HA sequence and epidemiology

Eleven hemagglutinin (HA) genes of seasonal (sH1N1 and H3N2) and 40 HA genes of pH1N1/09 influenza A viruses (IAVs) were included in this analysis. The fifty-one sequences of HA gene were translated into protein sequences and were then divided into two groups- “shared” strains versus “non-shared” strains which represented clustered transmission groups versus isolated introductions respectively. The criteria for a shared strain was 100% amino acid (aa) identity in two or more strains with date of collection within 5 days of each other, *or* from the preceding one in the cluster respectively. This would probably suggest that some kind of active transmission was going on. The two groups were then compared across various demographic characteristics such as age, gender, on-campus residence, course of study/faculty, and citizenship status.

### In-depth genomic analysis of pH1N1/09 viruses using comparative phylogenetic methods

Analysis was conducted on full-genomes of 34 pH1N1/09 viruses successfully sequenced in our study.

#### Phylogenetic analysis

For the whole genome concatenation Geneious R7 http://www.geneious.com [18] was used. The coding regions of gene segments PB-2, PB-1, PA, HA, NP, NA, M1, M2, NS1 and NS2 were concatenated using the software. The sequences were tested for any reassortment between strains using the RDP4 software [19]. Multiple alignment was performed with ClustalW in BioEdit [20]. The whole genomes were then used for the construction of phylogenetic trees using the best-fit nucleotide substitution model (TVM+Gamma+Invariant sites) determined by Modeltest 3.7 [21]. Bootstrapping with 1000 replicates was undertaken to assess the robustness of the phylogenetic trees. All the analyses were performed in the PAUP package [22].

#### Phylogeographic/ Bayesian Tip-Association (BaTS) analysis

BaTS (http://evolve.zoo.ox.ac.uk/Evolve/BaTS.html) was used to assess the degree to which the phylogeny of the viruses was related with the phenotypic traits [23]. The analysis was undertaken to determine the spatial dynamics of influenza infection in the localized university community of NUS. For the spatial component there were two categories: the location of the residence (‘On-campus’ or ‘Off-campus’) and faculty (‘Life Sciences’: Science, Nursing and Medicine faculties or ‘Non-Life Sciences’: Engineering, Business, Design and Environment, Computing, Arts and Social Science). The basis of separation into two faculties was the location on the campus. Life Sciences faculties are physically clustered in one region of the university whereas the Non-Life Sciences faculties are clustered together in altogether separate region of the university. The other phenotypic factors that were analysed for phylogenetic clustering were age (<25 or >25); gender (Male or Female); Singaporean (Yes or No). The BaTS program has a pre-requisite of Bayesian analysis as it uses the tree file with posterior probabilities that is generated in the BEAST v1.8 software [24]. The Bayesian analysis was performed using Bayesian Markov Chain Monte Carlo (MCMC) method with GTR+I+G substitution model along with estimated relaxed (uncorrelated lognormal) molecular clock. The analyses were performed with a time-aware Gaussian Markov Random Field Bayesian Skyride coalescent tree, with an Unweighted Pair Group Method with Arithmetic Mean-derived starting tree. All model parameters were given with relatively uninformative priori (default setting), except a uniform prior distribution for the mean substitution rate with initial value of 0.005 substitution per site per year and lower/upper limits of 0.0/1.0 [25, 26]. Analysis was performed with length of MCMC chain of 50 million to reach convergence and sampled at every 5000th generation. The analysis generated a total of 10000 samples for parameter estimates. Ten percent burn-in was applied for the analysis. This provided with a posterior set of trees which were then used to investigate each of the phenotypic traits listed earlier. Each trait was analysed as a binary character annotated into the posterior set of trees from the Beast analysis using the BaTS program. Two statistics (a) Association index (AI) and (b) Parsimony score (PS) were calculated to determine the phylogeny and trait association [27,28]. These two statistics determine the strength of phylogenetic clustering by place of isolation. A P value of ≤0.05 was considered statistically significant which means that the clustering was in fact associated with the trait under analysis and was not merely by chance.

#### Panoramic phylogenetic analysis

Further, these 34 genomes were analyzed in the context of 1518 global pH1N1/09 genomes retrieved from GenBank for the period from May 2009 to Sep 2009 using RAxML [29]. Trees were generated using the GTR substitution matrix with a GAMMA model of rate heterogeneity (all parameters were allowed to be estimated) and 1000 rapid bootstrap replicates were performed and examined with an ML search to find the best-scoring tree, which was subsequently visualized in MEGA 6 [30].

### Accession numbers

“Shared” strains (GenBank accession numbers for HA protein)- cluster S1: AGY41900-AGY41903, AGY41905, AGY41906, AGY41910, AGY41911, AGY41915, AGY41918- AGY41921, AGY41923, AGY41924, AGY41927- AGY41932, AGY41935, AGY41936, cluster S2: AGY41907, AGY41912, AGY41917, cluster S3: AGY41913, AGY41914, AGY41926, cluster S4: AGY41904, AGY41916) and “non-shared” strains (AGY41922, AGY41925, AGY41933, AGY41934, AGY41937-41, AGU69918, AGU69919, AGU69920, AGU69921, AGU69922, AGU69923, AHB72781, AHB72782, AGU69931, AGU69932, AGU69930. GenBank nucleotide accession numbers of 34 pH1N1/09 strains sequenced in this study: **PB2**: KP222548- KP222581; **PB1**: KP222582- KP222615; **PA**: KP222616- KP222649; **HA**: KF667880- KF667887, KF667890- KF667894, KF667896- KF667898, KF667900, KF667902- KF667910, KF667912, KF667914, KF667916- KF667921; **NP**: KP222650- KP222666, KF709407- KF709418, KF709420- KF709424; **NA**: KF695073- KF695080, KF695083- KF695101, KF695103- KF695109; **MP**: KP222667- KP222682, KP222684, KP222686- KP222694, KP222696, KP222698, KP222700- KP222705; **NS**: KP222706- KP222739.

## Results

### Preliminary analysis for related transmissions based on HA sequence and epidemiology

In the first analysis, using HA protein sequences and our own criteria for categorization of IAVs into shared and non-shared strains, we identified 31 ‘shared strains’ and 20 ‘non-shared strains’. This suggests at least some degree of transmission on-campus together with a limited amount of introductions. The 31strains in the shared group were all pH1N1/09 strains which could be further sub-divided into 4 clusters with 100% identical aa patterns. One of the clusters had 23 strains, two clusters had three strains each, and another cluster had two strains. The non-shared group had 9 distinct pH1N1/09 strains and 11 distinct seasonal influenza strains (sH1N1 and H3N2). Residence at a student hostel was identified as a risk factor for having a shared strain (Table 1).

**Table 1:** Association of epidemiological factors with clustered strains.

Epidemiological factor	Odds Ratio (95% CI)	P value
On-campus vs Off-campus residence	4.2 (1.2–14.9)	**0.02**
Age <25 vs Age >25 years	2.3 (0.6–8.9)	0.22
Gender	0.8 (0.2–2.7)	0.74
Singaporean vs Foreigner	1.7 (0.5–5.2)	0.38
Life Sciences vs Non-life sciences faculty	1.7 (0.5–5.9)	0.41

P value of ≤0.05 was statistically significant (shown in bold)

### In-depth genomic analysis of pH1N1/09 viruses using comparative phylogenetic methods

**Fig 1:**
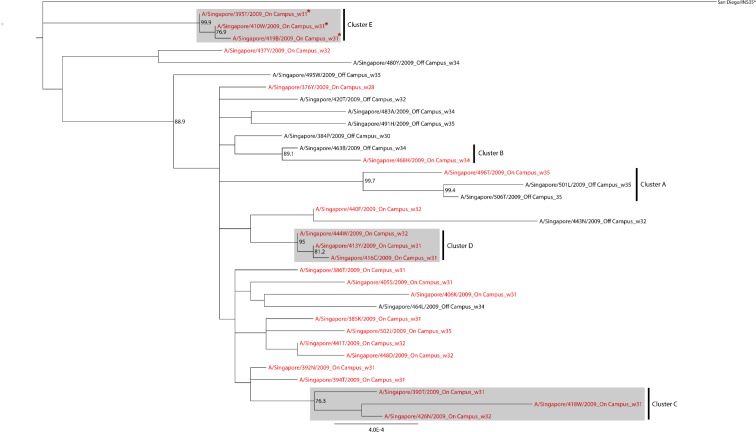
Maximum Likelihood phylogenetic tree of 34 concatenated genomes of pH1N1/09 viruses from NUS campus. Clade 6 viruses are marked with asterisk. Strain name is followed by residence status and week of isolation. On-campus sequences are in red font and Off-campus sequences are in black font. Clusters were identified with strong bootstrap support (>70%). Clusters with exclusively On-campus sequences are highlighted in grey color.

To ensure that our phylogenetic analysis of concatenated genomes is valid, we performed analysis for reassortment using RDP4 software and found no evidence of reassortment. We required the reassortment event to be consistently identified by at least three of the seven algorithms used in the RDP4 software. The second analysis which is phylogenetic analysis of full genomes of 34 pH1N1/09 viruses isolated in our study showed that both clade 6 and 7 were co-circulating on campus during the first pandemic wave in Singapore from July to August at NUS campus (Fig 1). It is possible that all the earlier clades might have been displaced by clade 6 and clade 7 viruses. From the phylogenetic tree (Fig 1), five well-supported clusters (boot strap value >70) of pH1N1/09 viruses from the university students and staff can be discerned. This clustering suggests that there was some degree of intra-university/on-campus transmission going on. The clusters were assigned names from A to E as shown in Fig 1. All the clusters (A, C, D and E) comprised three virus strains each, except one cluster (B), which had two strains. Of the five clusters, only cluster E had the three clade 6 virus strains while the rest had clade 7 viruses (Fig 1). Apart from clustered viruses, there were singleton viruses which may suggest independent viral introduction events. The phylogeny of the pH1N1/09 viruses was subsequently correlated with the epidemiological characteristics. The location of residence was compared among the various strains in each cluster. In three of the clusters: C, D and E, all the member strains were isolated from students residing on campus. This suggests possible intra-university transmission of influenza. However, in the other two clusters: A and B, both on-campus and off-campus strains clustered together. Universities represent a semi-closed setting and are themselves a small community, where the student interaction/mixing may happen at various places like hostels, classrooms, canteens, or while using the various university facilities. A closer look into the clusters with the aid of integrated phylogenetic and epidemiological data provided more insights into the transmission dynamics on campus. Cluster D contained three strains from students who reported sick on the same day, were from the same hostel, and even the same faculty which provide very strong evidence of intra-campus transmission. Furthermore, A/Singapore/413Y/2009 strain was isolated from a student who reported contact history with a sick neighbour in Malaysia. It is quite possible that he acquired infection in Malaysia and then spread it to other fellow students on campus. However, in cluster E, the first strain (according to the date of collection), A/Singapore/395T/2009, was isolated from a student from non-life sciences faculty and from a different hostel than the other two strains, A/Singapore/410W/2009 and A/Singapore/419B/2009. These 2 strains were isolated from students from life sciences faculty, who shared the same hostel and reported sick on the same day. This suggests the student with A/Singapore/395T/2009 strain could have possibly infected the students with strains A/Singapore/410W/2009 or A/Singapore/419B/2009 on campus but at a location other than hostel and classroom, for instance, at library, canteen or while using hostel facilities, suggesting some kind of spatial mixing on campus. Interestingly, there were some students who reported sick on the same day or one day apart and were from the same hostel and even same faculty but did not share highly similar strains.

To further strengthen the analysis and to clear the ambiguity associated with phylogenetic analysis, another test of phylogeny-trait associations was conducted using the BaTS software. Two statistics, AI and PS scores, were calculated for various demographic characteristics to explore the strength of clustering (Table 2).

**Table 2:** Results of phylogeny trait association for pH1N1/09 viruses.

Demographic Characteristic	Statistic	Observed value (95% CI)		Null value (95% CI)	P value
On vs Off campus residence	AI	0.76 (0.47–1.08)	1.69 (1.14–2.23)		0.009
	PS	7.08 (6.0–8.0)	8.90 (7.10–9.93)		0.04
Age <25 vs >25	AI	0.68 (0.42–0.96)	0.80 (0.48–1.2)		0.34
	PS	3.88 (3.0–4.0)	3.89 (3.06–4.0)		1.0
Male vs Female	AI	1.61 (1.06–1.9)	1.47 (1.06–1.99)		0.71
	PS	6.68 (6.0–7.0)	7.38 (6.0–8.0)		0.25
Singaporean vs Foreigner	AI	1.78 (1.40–2.16)	1.88 (1.19–2.6)		0.45
	PS	11.04 (10.0–12.0)	11.18 (8.91–13.04)		0.43
Life Sciences vs Non-life Sciences faculty	AI	0.50 (0.27–0.82)	1.38 (0.91–1.96)		0.02
	PS	5.87 (5.0–7.0)	7.33 (6.0–8.0)		0.05

AI- Association index; PS-Parsimony score; P value ≤0.05 was statistically significant, Life Sciences- Medicine, Nursing, Sciences; Non-life Sciences- Engineering, Computing, Business, Arts and Social science, Design and Environment.

The phylogeography of the viruses was analysed employing two characteristics: residence location and faculty. The residence location was annotated as ‘On-campus’ *or* ‘Off-campus’ and faculty was annotated as Life Sciences *or* Non-Life Sciences. The results showed that there was in fact some degree of clustering according to residence and this was not merely due to chance (AI P = 0.009; PS P = 0.04). There was also evidence of clustering with relation to faculty (AI P = 0.02; PS P = 0.05).

### Panoramic phylogenetic analysis

**Fig 2:**
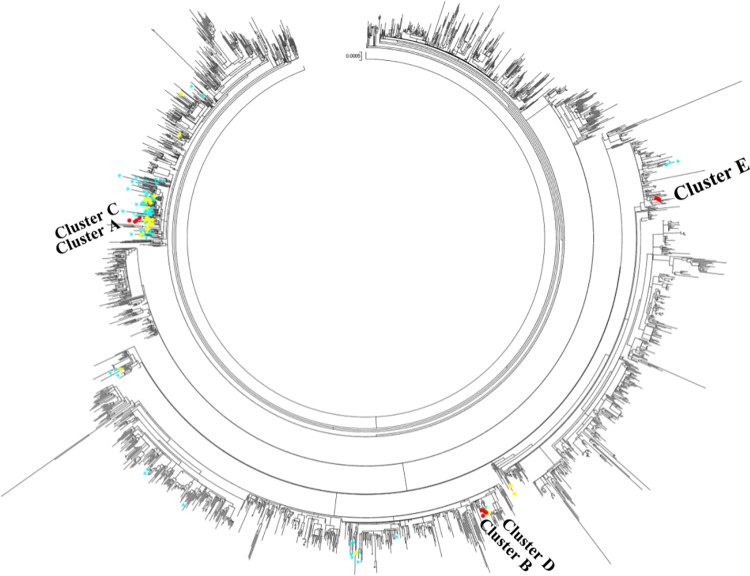
Maximum Likelihood phylogenetic tree of 1518 global genomes and 34 concatenated genomes of pH1N1/09 viruses. NUS sequences are in circles (red represent clusters and yellow others) and non-NUS Singaporean sequences are in blue triangles. Clusters were identified with strong bootstrap support (>70%). Trees were generated in RAxML using the GTR substitution matrix and GAMMA model of rate heterogeneity with 1000 bootstrap replicates. The best scoring tree was visualized in MEGA 6.

The third phylogenetic analysis showed (Fig 2) the estimated upper-bound of introductions on campus was 8 (NUS sequences separated from each other by non-NUS sequences as well as singleton lineages), and lower-bound of introductions was 4 (distinct lineages of pH1N1/09 viruses). This further implies that there was indeed influenza transmission going on on-campus.

## Discussion

This study was undertaken on a university campus to understand the spatial dynamics of spread and transmission of IAVs. There are some outbreak studies in university populations [31–33], but few phylogeographic studies in the literature.

We conducted three separate analyses to study the transmission dynamics of IAVs. The univariate epidemiologic analysis was based on the percentage of aa identity for HA protein sequences: ‘Shared’ & ‘Non-shared’ strains. Shared strains outnumbered non-shared strains, and all the shared strains were pH1N1/09 viruses. This suggests that the pH1N1/09 viruses were more transmissible than seasonal IAVs and this could be due to the viruses captured in the early phase of pandemic in this study when the virus had not undergone significant genetic drift. Residence at hostel was identified as an independent risk factor for having a shared strain. Clustering of identical strains in subjects staying on campus in hostels suggests that influenza transmission occurred on campus. While planning control strategies for influenza epidemics or mitigation strategies for pandemics, this demographic risk factor needs to be taken into consideration. Our sample size was too small for a meaningful multivariable analysis with multiple comparisons so in order to identify transmission clusters we also performed the genomic and phylogeogrphic analysis to confirm the epidemiological analysis. For genomic analysis, concatenated whole genome approach (34 genomes of pH1N1/09 viruses) was undertaken to determine the molecular epidemiology of influenza. The concatenation provides more concrete phylogenetic evidence than the single gene approach in understanding the complex microevolution of IAVs since genetic drift occurs in internal genes as well due to the error prone nature of low fidelity RNA polymerase. In our study, the majority of the circulating pH1N1/09 viruses belonged to either clade 6 or 7. The early pH1N1/09 viruses diversified into 7 clades as revealed by Nelson *et al*., over a short period since their appearance in 2009 [6]. This phylogenetic analysis provided some evidence of clustering of highly similar sequences suggesting intra-campus transmission. A strong evidence of clustering based on the residence and faculty of the students (P value for AI and PS <0.05) was supported by the phylogeographic analysis. Although a larger dataset is required to strongly substantiate the conclusion, this study still gives a good evidence of association of phylogeny with geography in a localized community of NUS. On the contrary, another study by Holmes *et al*., found no association between phylogeny and residence analysing 57 complete genomes of viruses on campus of the University of California, San Diego while there was intra-campus transmission [12].

The panoramic phylogenetic analysis provides a broader view of the process of transmission (Fig 2). The estimated upper-bound of introductions on campus was 8 and lower-bound of introductions was 4, which contributed to lower genetic diversification of virus and hence more clustering was seen. Clustering of the on-campus strains further supports the hypothesis that influenza transmission indeed occurred on-campus.

## Conclusions

Phylogenetic data showed evidence of well-supported geographical clustering of highly similar pandemic 2009 H1N1 influenza virus sequences with the majority from on-campus students suggesting some degree of intra-campus transmission. Furthermore, phylogeographic analysis strengthened the evidence of geographical clustering by providing statistically significant association of residence and faculty with clustering. Integration of molecular, epidemiological and statistical methods can help public health authorities to identify foci of transmission in localized communities such as universities. Identification of transmission hot spots can lead to more targeted intervention strategies, such as, closures of universities or campus-based quarantine and even potentially targeted chemoprophylaxis if there is sufficient evidence of intra-campus transmission.
